# An In-Depth Comparison of Latent HIV-1 Reactivation in Multiple Cell Model Systems and Resting CD4+ T Cells from Aviremic Patients

**DOI:** 10.1371/journal.ppat.1003834

**Published:** 2013-12-26

**Authors:** Celsa A. Spina, Jenny Anderson, Nancie M. Archin, Alberto Bosque, Jonathan Chan, Marylinda Famiglietti, Warner C. Greene, Angela Kashuba, Sharon R. Lewin, David M. Margolis, Matthew Mau, Debbie Ruelas, Suha Saleh, Kotaro Shirakawa, Robert F. Siliciano, Akul Singhania, Paula C. Soto, Valeri H. Terry, Eric Verdin, Christopher Woelk, Stacey Wooden, Sifei Xing, Vicente Planelles

**Affiliations:** 1 Veterans Administration San Diego Healthcare System, San Diego, California, United States of America; 2 Department of Pathology, University of California San Diego, La Jolla, California, United States of America; 3 Department of Infectious Diseases, Alfred Hospital, Melbourne, Australia; 4 Department of Medicine, University of North Carolina at Chapel Hill, Chapel Hill, North Carolina, United States of America; 5 Division of Microbiology and Immunology, Department of Pathology, University of Utah School of Medicine, Salt Lake City, Utah, United States of America; 6 Gladstone Institute of Virology and Immunology, University of California San Francisco, San Francisco, California, United States of America; 7 Department of Microbiology and Immunology, University of California San Francisco, San Francisco, California, United States of America; 8 Division of Pharmacotherapy and Experimental Therapeutics, Eshelman School of Pharmacy, University of North Carolina School of Medicine, Chapel Hill, North Carolina, United States of America; 9 Monash University, Melbourne, Australia; 10 Centre for Biomedical Research, Burnet Institute, Melbourne, Australia; 11 Department of Epidemiology, University of North Carolina at Chapel Hill, Chapel Hill, North Carolina, United States of America; 12 Department of Microbiology and Immunology, University of North Carolina at Chapel Hill, Chapel Hill, North Carolina, United States of America; 13 Department of Medicine, Johns Hopkins University School of Medicine, Baltimore, Maryland, United States of America; 14 Howard Hughes Medical Institute, Baltimore, Maryland, United States of America; 15 Department of Medicine, University of California San Diego, La Jolla, California, United States of America; Fred Hutchinson Cancer Research Center, United States of America

## Abstract

The possibility of HIV-1 eradication has been limited by the existence of latently infected cellular reservoirs. Studies to examine control of HIV latency and potential reactivation have been hindered by the small numbers of latently infected cells found *in vivo*. Major conceptual leaps have been facilitated by the use of latently infected T cell lines and primary cells. However, notable differences exist among cell model systems. Furthermore, screening efforts in specific cell models have identified drug candidates for “anti-latency” therapy, which often fail to reactivate HIV uniformly across different models. Therefore, the activity of a given drug candidate, demonstrated in a particular cellular model, cannot reliably predict its activity in other cell model systems or in infected patient cells, tested *ex vivo*. This situation represents a critical knowledge gap that adversely affects our ability to identify promising treatment compounds and hinders the advancement of drug testing into relevant animal models and clinical trials. To begin to understand the biological characteristics that are inherent to each HIV-1 latency model, we compared the response properties of five primary T cell models, four J-Lat cell models and those obtained with a viral outgrowth assay using patient-derived infected cells. A panel of thirteen stimuli that are known to reactivate HIV by defined mechanisms of action was selected and tested in parallel in all models. Our results indicate that no single *in vitro* cell model alone is able to capture accurately the ex vivo response characteristics of latently infected T cells from patients. Most cell models demonstrated that sensitivity to HIV reactivation was skewed toward or against specific drug classes. Protein kinase C agonists and PHA reactivated latent HIV uniformly across models, although drugs in most other classes did not.

## Author Summary

HIV establishes a state of latency in vivo and this latent reservoir, although small, is difficult to eradicate. To be able to better understand this state of latency, and to develop strategies to eliminate it, many groups have developed in vitro models of HIV latency. However, notable differences exist among cell model systems because compounds that reactivate latent HIV in a particular system often fail to do so uniformly across different models. To begin to understand the biological characteristics that are inherent to each HIV model of latency, we compared the response properties of five primary T cell, four J-Lat cell models and those obtained with patient-derived infected cells. A panel of thirteen stimuli that are known to reactivate HIV by defined mechanisms of action was selected and tested in parallel in all models.

## Introduction

The possibility to achieve HIV eradication has been limited, at least in part, by the existence of latently infected cellular reservoirs [1]–[3]. The major known cellular reservoir is established in quiescent memory CD4+ T cells, providing an extremely long-lived set of cells in which the virus can remain transcriptionally silent [1]–[3]. Reactivation of latent viruses followed by killing of the infected cells has been proposed as a possible strategy (“shock and kill”) to purge the latent reservoir [4].

Studies to examine the control of HIV latency and potential reactivation have been hindered, however, by the small numbers of latently infected cells *in vivo* and the absence of known phenotypic markers that can distinguish them from uninfected cells. In this setting, cell-line models of latency have been very useful due to their genetic and experimental tractability. Major conceptual leaps have been facilitated by the use of latently infected T cell lines [5]–[10], including the ability to conduct genetic screens [11]. On the other hand, latently infected cell lines are limited by their cycling nature and inherent mutation in growth controls, and the clonal nature of the virus integration sites. Such transformed cell lines lack the ability to differentiate and naturally oscillate between phases of quiescence and active proliferation in response to biological signals. Because of these limitations, a number of laboratories have recently developed primary cellular models of HIV-1 latency that capitalize on specific aspects of the T cell reservoir, found *in vivo* (reviewed in references [12]–[14]). These newer models afford investigators the ability to easily and rapidly study proposed mechanisms governing latency and to evaluate novel small molecule compounds for induction of viral reactivation.

One significant complication, associated with the present variety of available latency models, is that notable differences exist among the cell model systems. Disparities relate to: the T-cell subsets being represented; the cellular signaling pathways that are capable of driving viral reactivation; and the genetic composition of the viruses employed, ranging from wild-type to functional deletion of multiple genes. Additional differences reside in the experimental approaches taken to establish latent infection in these primary cell models, which involve either infection of activated cycling cells that are later allowed to return to a resting state [15]–[19], or direct infection of quiescent cells [20], [21]. Because of such system variables, screening efforts in specific cell models with identified drug candidates for “anti-latency” therapy often fail to reactivate HIV uniformly across the different models. Therefore, the activity of a given drug candidate, demonstrated in a particular cellular model, cannot predict reliably the activity that will be seen in other cell model systems or in infected patient cells, tested *ex vivo*. The current situation in this research field represents a critical knowledge gap that is adversely affecting our ability to identify promising treatment compounds and their associated molecular mechanisms and is hindering the advancement of drug testing into relevant animal models and ultimately, human clinical trials.

The present work represents a broad collaborative effort to compare and contrast induction of HIV reactivation across a battery of well-characterized cell models of viral latency, employing a highly coordinated and standardized testing approach. This work is based on the premise that it is unlikely that a single *in vitro* cell model can completely recapitulate the biological properties of the latent reservoir *in vivo*, let alone reflect accurately the response characteristics of infected patient cells *ex vivo*. Therefore, it is important to define both the common and unique properties among the available cell models of HIV latency in order to design a rational approach to employ such models in the identification of valid candidate drugs to induce HIV reactivation.

Examples of how such an approach also can inform the underlying mechanistic actions of experimental compounds are available in the field. For instance, in the latency model developed by Bosque *et al.* [15], the derived central memory CD4^+^ T cells (T_CM_) are highly responsive to stimuli that activate the nuclear factor of activated T-cells (NFAT); on the other hand, virus reactivation from J-Lat clones [8] tends to be highly responsive to stimuli that activate the nuclear factor kappa of B cells (NFκB), such as protein kinase C (PKC) activators and tumor necrosis factor-alpha (TNF-α). Although the use of these two model systems would predictably yield different types of hits during a compound library screen, it is important to note that known compounds, which signal through either of these activation pathways, are capable of reactivating HIV replication in latently infected CD4+ T cells from patients *ex vivo*, and by inference, perhaps *in vivo*.

To begin to understand the biological characteristics that are inherent to each model of HIV-1 latency, we compared the properties of six models (Table 1), to those obtained with a standard viral outgrowth assay using patient-derived infected cells [1], [22]. As no specific denominations have been assigned to these models, we have for simplicity referred to them by the name of the senior investigator in whose laboratory they were developed. They included the following (details are provided within the Methods section):

**Table 1:** Properties of the models used in this study.

Model/Cell type	Source of T-cells	Cell cycle status upon infection	Phenotype during latency	Virus/vector	Readout upon reactivation
Greene	Primary CD4+ T cells	Resting	T_CM_, T_TM_	NL4-3 (WT reporter)	Luciferase (RLU)
Lewin	Primary, resting CD4+ cells	Resting	CCR7+/CD45RO+/HLA-DR−/CD25−/CD69−	NL4-3 (WT)	Soluble RT activity
Patient Cells/QVOA	Primary, resting CD4+ T-cells from infected patients	NA	CD25−/DR−	Endogenous	IUPM (limiting dilution)
Planelles	Primary naïve CD4+ T-cells	Dividing	CCR7+, CD27+, CD45RO+, CD25^low^ CD69−	HIV-1Δenv or HIV-1ΔenvΔnefGFP	%GFP+ or % IC-Gag+ cells
Siliciano	Primary CD4+ T-cells	Dividing	CD45RO+, CD62L+ CCR7−	NL4-3Δ6-drEGFP	% GFP+ cells
Spina	Primary CD4+ T cells	Resting	Mixture of T_N_, T_cm_, T_EM_, T_E_	HIV-1 NL4-3 (WT)	Tat mRNA copies
Verdin	Jurkat-derived clones	Dividing	NA	HIV-1 R7 (GFP)	% GFP+ cells

_CM_: CCR7+, CD27+, CD45RO+, CD25^low^ CD69−; T_TM_: CCR7−, CD27+, CD45RO+, CD25^low^ CD69−; T_N_: CCR7+, CD27+, CD45RO−; T_EM_: CCR7−, CD27−, CD45RO+. T

The Greene laboratory model [23] is a modification of the original O'Doherty model of latency [20] and establishes HIV infection directly in quiescent primary CD4+ cells, using spinoculation delivery of virus. Replication-competent NL4-3 reporter virus is used, which contains Luciferase in the *nef* reading frame (Δ*nef*/luciferase). After a short 3 day-culture, induction of provirus activation from latency is performed in the presence of integrase inhibitor to prevent viral spread and the contribution of any unintegrated viral species. Quantification of HIV replication by Luciferase expression is population-based. While only approximately 5–10% of the culture contains latently infected cells, this assay permits the generation and analysis of test compounds within 6 days.

The model developed by Lewin and colleagues uses exposure of primary resting CD4+ T cells to chemokines that bind to receptors CCR7, CXCR3 or CCR6 to effectively establish infection with wild-type NL4-3 virus [21], [24]. Incubation with the chemokines does not cause significant cellular activation, but induces changes in the cellular actin cytoskeleton, which allows for efficient virus nuclear localization, integration, and establishment of latent infection [24]. Treatments to reactivate virus are followed by co-culture with amplifying feeder cells. Productive HIV replication is determined on a total population basis by quantification of soluble reverse transcriptase (RT) activity released into culture.

The Planelles model [14], [15] establishes viral latency in cultured primary CD4+ T cells that have been differentiated by TCR stimulation in the presence of TGF-β, and αIL-4 and αIL-12 monoclonal antibodies into a non-polarized subset, representative of central memory cells (T_CM_) [14], [25]. Spinoculation with a packaged *env* defective NL4-3 clone establishes a single round of infection in the majority of the cells that transition into latency. Induced reactivation of HIV is monitored on a per-cell basis, using staining and flow cytometry detection for intracellular Gag (p24) expression.

The Siliciano model [17] uses a two-step derivation of latency in cultured primary CD4+T cells, isolated from peripheral blood. In the first step, cells are TCR stimulated, transduced with the EB-FLV lentiviral vector, for constitutive expression of Bcl-2, expanded in culture with IL-2 and allowed to return to a resting state. In the second step, the cells are reactivated and infected with a trans-packaged, replication defective NL4-3 GFP-reporter virus clone (NL4-3-Δ6-drEGFP). After 3–4 weeks of culture, the GFP-negative cell subset, expressing a quiescent effector memory cell (T_EM_) phenotype, is isolated by flow cytometry sorting. Approximately, 2–6% of the recovered cells carry latent HIV infection. Reactivation of virus replication is tracked by GFP expression, on an individual cell basis.

The Spina model (unpublished results; manuscript submitted) is based on early work demonstrating that HIV-1 can establish infection directly in resting primary CD4^+^ T lymphocytes *in vitro* [26], [27], and on recent work showing that during acute HIV infection in a heterogeneous population of primary CD4+ T cells, undergoing varying degrees of cell activation, viral latency is established early and preferentially in non-dividing and minimally activated cells. This model uses the experimental approach of deriving latent NL4-3 infection (wild-type) in non-dividing “bystander” cells during brief co-culture with autologous productively infected, proliferating cells. When the quiescent bystander cell population is isolated from co-culture, the latently infected subset ranges from 1 to 12% cells containing integrated HIV DNA, and 0.5–5% cells with inducible provirus, as measured by expression of intracellular Gag. Latent infection is found in all of the major phenotypic subsets of T cells: naïve, central memory and effector memory. After incubations with experimental compounds, reactivation of virus replication is measured on a population basis, through quantification of *tat* mRNA by RT-qPCR.

Verdin and colleagues have generated a number of Jurkat cell line-derived clones, bearing latent HIV-1 in single integration sites, that were engineered to express GFP in lieu of *nef* [8] (J-Lat). J-Lat cells have been used in numerous studies that have contributed a wealth of knowledge in the area of viral latency. In contrast to several other models of HIV latency in cell lines, where mutations are present in the HIV *tat* gene or the TAR element, the J-Lat cell model contains wild-type *tat* and TAR. Three J-Lat clones established in the Verdin laboratory, 6.3, 8.4, 11.1 and one clone generated by the Greene laboratory, 5A8, have been included in this comparison. J-Lat 5A8 was derived by specifically selecting for cells that would be more responsive to αCD3/αCD28 co-stimulation than the parental J-Lat line [28]. Under untreated basal conditions, little or no GFP expression is detected. However, reactivation of latent provirus is readily monitored by flow cytometry analysis of GFP expression.

Results obtained with the above cell models were compared to results obtained in quantitative viral outgrowth assays (QVOA; patient cell assay) performed in the Margolis laboratory, with resting CD4+ T cells obtained from the leukopheresed peripheral blood of aviremic, ART-treated HIV-infected patients. This assay, as first described by three laboratories [1]–[3], was later modified to its present design [22]. Following negative selection, resting CD4+ T cells are incubated with integrase and reverse transcriptase inhibitors to ensure the decay of any HIV genomes in the state of pre-integration latency [29]. The cells are exposed briefly to test compounds, and then plated in replicate microwells in a terminal-dilution assay and cultured with PHA-stimulated, allogeneic irradiated peripheral blood mononuclear cells (PBMC) from a sero-negative donor, and rIL-2. After 19 days, the microcultures are scored for virus replication by soluble p24 production, and the number of cells containing replication-competent HIV is expressed as infectious units per million CD4+ T cells (IUPM).

Induction of viral reactivation across all cell models was assessed using a selected common panel of stimuli that are known to function by distinct and defined mechanisms of action. The panel included 13 treatments (Table 2) that modulate T cell processes such as T-cell receptor engagement, protein kinase C (PKC) activation, calcium influx, cytokine signaling, histone deacetylation, and release of P-TEFb from the HEXIM/7SK RNP complex. This study was designed to answer the following questions: 1) are certain models of latency biased towards or against particular cell signaling pathways; 2) can stimuli be identified that work uniformly in multiple models; 3) can a central uniting theme or a single signaling pathway be responsible for control of viral latency; and 4) can a model or limited group of models predict experimental drug activity in authentic latently infected cells from patients?

**Table 2:** List of stimuli used in this study and their corresponding signaling pathways.

Stimulus	Physiologic Activity	Signaling Axis
**αCD3+αCD28 PHA**	TCR engagement	Lck/Calcineurin/NFAT and PKC/NFκB
**PMA Prostratin Bryostatin**	PKC activation	PKC/NFκB and PKC/MAPK
**PMA+Iono.**	PKC activation and Ca^++^ influx	PKC/NFκB; PKC/MAPK and calcineurin
**TNF-α**	TRAF recruitment	NFκB/AP-1
**IL-7+IL-2**	γc-receptor engagement	JAK/STAT and PI3K/AKT/NFκB
**SAHA MRK-1 MRK-11**	HDAC inhibition	Chromatin remodeling and activation of transcription (not gene-specific)
**HMBA**	Dissociation of P-TEFb from 7SK-RNP	P-TEFb (not gene specific)
**Ionomycin**	Ca^++^ influx	Calcineurin

## Results

**Figure 1:**
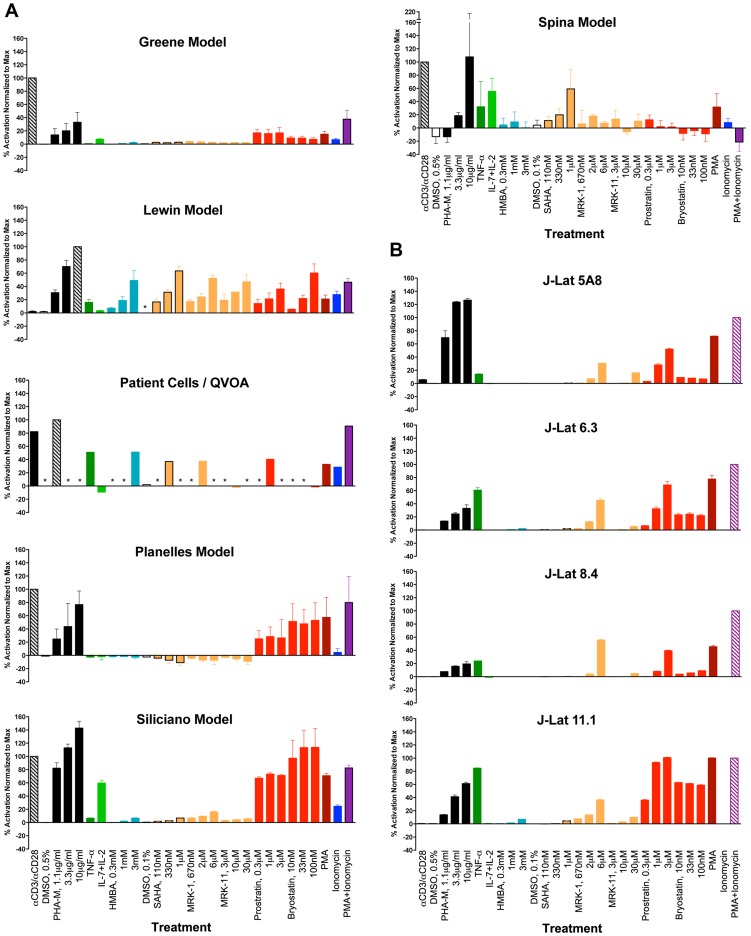
Graphic summary of the ability of each compound to activate HIV within each cell model: (A) primary CD4 T cell models and patient cell outgrowth assay (QVOA), and (B) J-Lat T cell line clones. Each compound and concentration tested is listed on the X-axis. In the primary CD4 cell models, each compound was tested using cells from 2, 3 or 4 different donors and in duplicate or triplicate with cells from each donor (See Methods Section for details). For the QVOA, results from the limiting dilution cultures from 3 patients were pooled to calculate one common IUPM (infectious units per million cells) value which was then normalized to that obtained with PHA. With the J-Lat clones, experiments were performed in triplicate. Asterisks represent “not done”.

Thirteen stimuli shown in Table 2 were chosen on the basis of their known or proposed activities in reactivating latent HIV-1 in various systems. For primary cell assays, experiments were performed with cells from three different donors, and replicate samples (duplicate or triplicate) were used for each treatment variable tested (refer to Methods Section for details). For J-Lat clones, experiments were performed in triplicate for each clone. Figure 1, panels A and B depict the average responses (mean +/− SEM) obtained with each of the cell models and the patient cell outgrowth assay. In all cases, the stimulus providing maximal reactivation response was used as a reference and assigned a 100% value, and the results from all other stimuli were normalized as a percentage of the maximal response (Figure 1). Within each individual experiment (*e.g.*, donor cells), the untreated baseline value was first subtracted from each treatment response value, prior to the normalization step. The average relative response was then calculated across all experiments (donors) for each stimulus tested. While transformed and primary cell models could be tested at three concentrations of each stimulus, assays with patient cells/QVOA were only performed at a single drug concentration due to limiting cell numbers. Therefore, two comparisons were performed: one which included all concentration points for each drug, and did not include patient cell assay data; and a second one in which a single concentration point was considered, to provide an analysis that could include patient cell results.

The maximal response for all primary models, except for the Lewin model and the patient-cell outgrowth assay, was obtained with αCD3+αCD28 antibody stimulation. In the Lewin model and the QVOA, PHA was the stimulus yielding a maximal response. The maximal response in the four J-Lat clones was obtained with PMA+Ionomycin. In all the J-Lat clones, except 5A8, CD3 surface expression is normally downregulated in culture (E.V., W.C.G., unpublished data). CD3 downregulation makes these cells unresponsive to αCD3/αCD28 antibody stimulation, although they remain responsive to PHA (most likely through engagement of the CD2 receptor). An additional representation of the data is shown in Figure S1, where, for each treatment, only the concentration of compound that was most active is represented.

### T-cell receptor engagement

T-cell receptor engagement is effectively mimicked by the binding and cross-linking of antibodies against CD3ε, one of the signal transduction subunits in the CD3 complex [30] and the co-stimulatory molecule, CD28 [31].

Phytohemagglutinin (PHA-M) is a lectin that binds to carbohydrate moieties on surface glycoproteins. PHA is a polyclonal mitogen for T cells. Both PHA and αCD3/αCD28 antibody treatments stimulate signaling cascades that encompass TCR/LCK/p38 activation leading to calcineurin and NFAT activation, as well as PKC stimulation leading to NFκB activation. Incubation with αCD3/αCD28 antibody-coated beads produced strong responses in all primary cell models, with the exception of the Lewin model (Figure 1A). In contrast, all J-Lat clones, except 5A8 were completely unresponsive to αCD3+αCD28 incubation (Figure 1B). The response of J-Lat 5A8 cells after stimulation by αCD3+αCD28 coated beads, although detectable, was lower than that displayed by most primary cell models. However, the levels of stimulation can be improved using plate-bound αCD3 and free αCD28 antibodies, if so desired (D.R. and W.C.G., data not shown). Moreover, these cells are highly responsive to PHA, which indicates that these cells contain an intact signaling pathway downstream of TCR engagement.

PHA reactivated latent viruses in all primary cell models and in the J-Lat clones, although with variable efficiency (Figure 1, Panels A and B). Therefore, the lack of responsiveness of J-Lat clones and of cells in the Lewin model to αCD3+αCD28 antibody treatment cannot be attributed to a lack of signaling mediators, since these cells respond to PHA through a highly similar signaling pathway.

### Activation through protein kinase C

PKC is a family of ten kinases that are activated by phorbol esters [32]. In general, phorbol esters promote activation and differentiation of monocytes and monocytoid cells, as well as potent T-cell activation. Three PKC agonists were tested, namely PMA, prostratin (both phorbol esters); and bryostatin-1 (a cyclic polyketide). PKC agonists activate the DAG-PKC-NFκB signaling pathway. PMA has long been used as a T-cell mitogen. PMA was tested at 2 nM in primary cell models and 16 nM in J-Lat clones. At these concentrations, PMA elicited maximal or near-maximal responses in J-Lat cells, except in clone 8.4. Responses to PMA were near maximal in the Planelles and Siliciano models; the rest of the primary cell models and the QVOA also showed viral reactivation in response to PMA, although at more modest levels.

Prostratin is a unique phorbol ester in that it induces potent T cell activation signals but, unlike PMA, is not tumorigenic. The ability of prostratin to induce T-cell activation through PKC, without tumor promoting ability, has made prostratin the subject of studies for its possible use as an inductive adjuvant therapy in the context of anti-retroviral therapy (ART) [33]. Another unique property of prostratin is that, despite being able to reactivate latent HIV-1, it exerts an inhibitory effect on active HIV-1 replication through downregulation of CD4 [34], [35]. The relative reactivation efficiencies observed in response to prostratin were similar to those obtained with PMA treatment. Thus, the models with the highest responses to PMA (J-Lat 6.3 and 11.1 clones, and Siliciano and Planelles models) showed the highest responses to prostratin as well. Conversely, poor to intermediate responses to PMA, observed in the Greene, Lewin and Spina models, and the quantitative patient-cell outgrowth assay (QVOA) were paralleled by similar responses to prostratin (Figure 1, Panels A and B). In the specific case of the Greene model, it has been observed that only a minority of cells, about 5%, respond to PMA, although the reasons for this observation are unknown.

Bryostatins are a family of natural products found in several species of bryozoans. Bacterial symbionts of the bryozoan species are thought to be responsible for bryostatin synthesis (reviewed in [36]). Bryostatins bind to the diacylglycerol-binding region within the C-1 regulatory domain of PKC. Bryostatin-1 was recently shown to reactivate latent HIV-1 in vitro in monocytoid and lymphoid cell line models of latency [37] and was approximately 1,000-fold more potent than prostratin. More recently, DeChristopher and colleagues achieved the chemical synthesis of several analogs of bryostatin-1, which demonstrated potent activity in J-Lat cells [38]. Bryostatin-1 was very potent in J-Lat clone 11.1, but had only modest activity in the other J-Lat clones (Figure 1B). In primary cell models, bryostatin-1 induced maximal response in the Siliciano model, and about half-maximal responses in the Lewin and Planelles models. However, the Greene and Spina models, and the patient cell outgrowth assay, showed very low to non-detectable responses to bryostatin-1 (Figure 1A).

### PKC stimulation in combination with a calcium ionophore

A commonly utilized T-cell activation regimen in the laboratory, which mimics the signaling pathway used in TCR engagement, is the combination of PKC activation via PMA along with the calcium ionophore, Ionomycin, which bypasses the requirement for both CD3/TCR and CD28 receptor engagements. Signaling downstream of TCR engagement involves the formation of inositol triphosphate, which triggers an increase in the intracellular Ca^2+^ concentrations, which in turn activate the phosphatase, calcineurin. Calcineurin then dephosphorylates cytoplasmic NFAT transcription factor, which translocates to the nucleus. A combination of PMA and Ionomycin induced vigorous viral reactivation in most cell models tested, but not in the Spina model. Viral reactivation in response to PMA+Ionomycin was generally increased when compared to that of PMA alone, with the exception of the Lewin and Spina models (Figure 1, panels A and B). Unexpectedly, PMA+Ionomycin stimulation of primary T-cells in the Spina model caused inhibition of Tat mRNA transcription, the readout in this assay, to below initial basal levels (Figure 1A). It has been reported previously that PMA induction of HIV replication can be Tat-independent [39]; and in this case, the combination with Ionomycin appeared to actually suppress Tat transcription at 24 hrs. following stimulation. In the patient cell outgrowth assay/QVOA, PMA+Ionomycin produced a strong reactivation response that was higher than that observed with each compound alone.

### Cytokine stimulation

Previous reports showed that incubation with IL-7, alone [40] or in combination with IL-2 [41] can reactivate latent HIV-1 in resting CD4+ T cells isolated from infected individuals. IL-7 also reactivated latent HIV-1 in thymocytes in a SCID-hu mouse model of HIV latency [42] and in cultured T_CM_ in the Planelles model [43]. In the Planelles model, IL-2+IL-7 stimulation of latently infected cells was previously shown to be inefficient (10–20% of the reactivation obtained with αCD3/αCD28) and to promote division of infected cells in the absence of viral reactivation [43]. Responsiveness to IL-7, or IL-2+IL-7 stimulation is physiologically relevant as these cytokines, along with IL-15, are known to drive the homeostatic proliferation of memory T cells in vivo [44]. A recent study found that IL-7, when administered to HIV-1 infected patients undergoing ART, promotes viral persistence by enhancing residual levels of viral production and inducing proliferation of latently infected cells without reactivation [45]. Robust responsiveness to IL-2+IL-7 was observed in the Siliciano and Spina primary cell models, and minimal activity was observed in the Greene model. Cells in the Lewin and Planelles models and the patient cell outgrowth assay responded poorly or not at all (<5% of maximal); whereas, cells in the Greene model exhibited a weak response. It is interesting to note that IL-7 used alone at 25 ng/ml induced robust reactivation in the Lewin model [46]. J-Lat cells failed to reactivate virus in response to IL-2+IL-7 stimulation. Jurkat cells, the parental tumor cell line from which J-Lat clones were derived, are IL-2-independent for their growth and survival, do not express the high-affinity IL-2 receptor, CD25 [47], [48], and express low levels of the IL-7 receptor alpha [49].

TNF-α is a potent inducer of viral gene expression in certain tumor cell lines harboring integrated, latent HIV-1, through the activation of NFκB [5], [8], [50], [51]. As previously reported, TNF-α treatment activated virus expression in J-Lat cells, especially in clones 6.3 and 11.1 (Figure 1B). However, among the primary cell models, TNF-α failed to induce any detectable viral reactivation in the Greene and Planelles models and showed only minimal activity in the Lewin and Siliciano models. In contrast, the patient cell outgrowth assay responded robustly to TNF-α, and cells in the Spina model showed an intermediate response.

In order to better understand the responsiveness, or lack thereof, to TNF-α, we analyzed the levels of TNF-R in primary and Jurkat cells. We isolated bulk PBMC from two donors, selected memory CD4+ cells using CD45RO expression, and then stained the cells for CCR7, CD27 and the TNF-α receptor. These experiments showed that none of the freshly selected memory subsets tested (specifically, T_CM_, T_EM_ and transitional memory T cells, T_TM_) expressed detectable levels of the TNF-α receptor (Figure S2). TNF-R expression was extremely low in cultured T_CM_ from the Planelles model (Figure S2). In contrast, J-Lat 10.6 cells expressed high levels of TNF-R (Figure S2). HIV reactivation in response to TNF-α in vitro and in vivo is likely linked to whether cells under the specific culture or physiological conditions upregulate the expression of the TNF-α receptor.

### HMBA

Hexamethylene bisacetamide (HMBA) is a hybrid bipolar compound that induces differentiation and apoptosis in transformed cell lines in culture [52], [53]. HMBA was shown to activate HIV transcription in vitro [7], [54], to reactivate latent HIV in vitro [19], [55] and to reactivate HIV in primary cells from aviremic, infected patients [56]. The activity of HMBA on HIV transcription is a result of its ability to induce dissociation of P-TEFb from the inhibitory 7SK ribonucleoprotein complex [19], [55].

HMBA treatment had significant reactivation activity in the QVOA and the Lewin model, but demonstrated little to no activity in the rest of the primary cell models and J-Lat clones tested (Figure 1, panels A and B).

### Histone deacetylase inhibitors

The “histone code” model states that a variety of covalent, post-translational modifications (PTM) on histone tail residues regulate the interaction of transcriptional regulators with chromatin to determine gene expression levels. The nature and localization of such post-translational modifications is broad, and their ability to act in a combinatorial manner provides an attractive model for how a finely tuned regulation can be effected. Histone code modifications include acetylation, phosphorylation, methylation, ubiquitination and sumoylation, among others [57], [58]. Acetylation of lysine residues in histone tails can have two important effects on chromatin organization (reviewed in [58]). First, this PTM results in neutralization of a basic charge on the lysine residue, which results in disruption of histone contacts with other histones and with DNA, diminishing the degree of compaction of the local chromatin. Second, proteins containing a specialized domain known as bromodomain specifically recognize acetylated lysine residues and then trigger downstream regulatory effects. Acetylation of histones is regulated by the concerted action of HATs and HDACs. Acetylated histones have long been associated with actively transcribed genes [59] and, therefore, inhibitors of HDAC (HDACi) are considered as general activators of transcription. Two main categories of HDACs have been described: Class I (HDAC 1, 2, 3 and 8), and Class II (HDAC 4, 5, 6, 7, 9, 10 and 11). Inhibition of Class I, but not Class II, HDACs has been shown to induce reactivation of latent HIV [60], [61].

Suberoylanilide hydroxamic acid (SAHA; also known as vorinostat) is a pan-HDAC inhibitor that targets both Class I and Class II HDACs, and can induce reactivation of HIV in models of HIV latency [11], [62]–[65], and in resting cells from ART-treated, aviremic HIV-infected patients [65]–[67], although it failed to induce reactivation in patient cells in another study [68]. Recently, a single administration of SAHA to ART-treated, aviremic patients was shown to induce global cellular acetylation and increases in viral RNA in resting CD4+ cells from these patients [69].

To test the ability of HDAC inhibitors to reactivate latent HIV in the various models of latency, we utilized three such inhibitors, provided by Merck Research Laboratories. SAHA potently blocks the Class I HDACs (*i.e.*, 1, 2, 3, and 8) and has modest activity against Class II HDACs (*i.e.*, 6, 10 and 11). MRK-1 is a selective inhibitor of the Class I HDACs (*i.e.*, 1, 2 and 3) and HDAC6 (Class II); whereas, MRK-11 selectively blocks Class II HDACs (*i.e.*, 4, 5, 6 and 7) and HDAC8 (Class I) [60].

SAHA was moderately potent in the Lewin and Spina cell models and QVOA, but was marginally active or inactive in the rest of the primary cell models and the J-Lat clones. The activity profile of MRK-1 was similar to that of SAHA in the primary models, showing the best activity in the Lewin cell model and the patient cell outgrowth assay. All the J-Lat clones had modest responses to MRK-1, which contrasted with the poor activity seen with SAHA in these cells (Figure 1B). The differences between SAHA and MRK-1 responses could, potentially be explained by the slightly different specificities of these HDAC inhibitors.

In general, MRK-11 was inactive or minimally active (<20% response) in the QVOA and all J-Lat and primary cell models, except in the Lewin model, where it exhibited close to 50% activity. Cells in the Lewin model are unique in this study, in that they are very sensitive to viral reactivation by both Class I and Class II HDAC inhibitors. In contrast, other models tested are either insensitive to HDACi or show sensitivity to Class I inhibitors but not to Class II.

### Similarities between models with respect to their response to activating compounds

**Figure 2:**
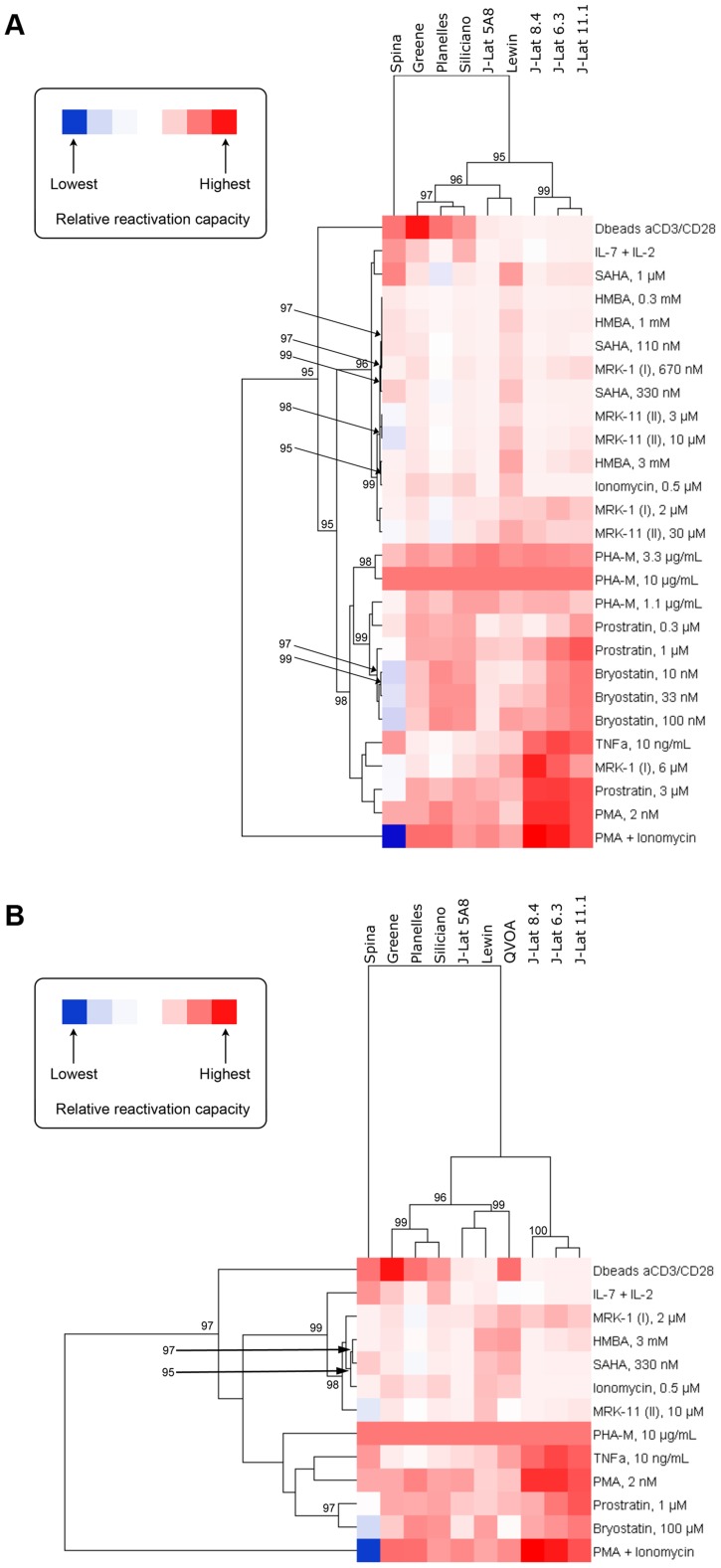
Heatmap visualization of the ability of each compound to activate HIV within each model when excluding (A) and including (B) data from the QVOA model. A reduced set of compounds was analyzed in (**B**) since not every compound was run at every concentration in the QVOA. The clustergram at the left of each heatmap reflects the relationships between compounds based on their ability to activate HIV across compounds. Since cells in all models responded to PHA with high strength, ranking was normalized within each model to the response to PHA at 10 µg/mL and, therefore, all models display in the heatmap the same relative responsiveness to this treatment. The clustergram at the top of each heatmap reflects the relationship between each model based on their response to compounds. Clustergrams were created by calculating Euclidean distances and then clustering distances using the average linkage method. The numbers at the nodes of clusters are AU p-values where 95% represents a *p*-value cut-off of 0.05 and only values 95% or greater are depicted. Red cells in the heatmaps reflect HIV activation whereas blue or blank cells indicate that the compound did not effectively activate HIV.

The relationship between models based on the ability of compounds to activate latent HIV within each model was investigated by hierarchical clustering and heatmap visualization (Figures 2A and 2B). Two comparisons were performed. First, all the cell models for which data was available for all compounds and at all concentrations were compared (Figure 2A). This comparison excluded the patient cell outgrowth assay for which data for only certain concentrations of activators were available. In the second comparison, all models were included but only those concentrations that were universally tested were included (Figure 2B). In both comparisons, reactivation values obtained with PHA at 10 µg/ml were used as a reference, to which all other reactivation values were normalized to.

Both comparisons yielded strikingly similar results. Three significant clusters of models were identified, with one robust outlier, the Spina model. The Lewin and J-Lat 5A8 clustered very close in both comparisons (Figures 2A and 2B), with the patient cell assay/QVOA being the next closest to those two (Figure 2B). Therefore, the first subcluster is defined by the Lewin, J-Lat-5A8 and QVOA models. The second subcluster is defined by the Planelles and Siliciano models, closest to each other, and the Greene model. The first two subclusters have a close association with each other, that separates them from the three remaining J-Lat clones (8.4, 6.3 and 11.1), which form the third and more distant subcluster.

This clustering conforms to what would be expected biologically with the majority of primary cell models clustering together and the majority of cell line models clustering separately, with the exception of J-Lat 5A8, which clusters among the primary models. In addition, this clustering pattern was largely maintained when the QVOA data was included and a reduced compound set analyzed (Figure 2B). Since all primary cell models clustered together, this suggests that the resting phenotype of these models compared with the proliferating phenotype of J-Lat cells may influence the responsiveness to different agents. The QVOA model appears to cluster robustly with the Lewin model and the J-Lat 5A8, suggesting that these two models may represent the best proxy currently available for the activation capabilities of compounds when analyzing cells from HIV-infected subjects. However, this interpretation should be treated with caution as the clustering in Figure 2B, when the QVOA data was included, was performed with a reduced compound set and may not be as robust as the analysis that included all compounds at all concentrations (Figure 2A).

### Similarities between activating compounds with respect to their activity across models

The relationship of compounds to each other, based on their ability to activate HIV across the different models, was also investigated by hierarchical clustering and heatmap visualization (Figures 2A and 2B). The first analysis (Figure 2A) revealed that PMA+Ionomycin and, separately, αCD3+αCD28 antibody stimulation represented treatments that were strong outliers. The rest of the compounds then fell into one of two significant major clusters. The first cluster contained the majority of the HDACi, but also IL-7+IL-2 treatment, Ionomycin, and HMBA. The second cluster contained all concentrations of the PKC activators (*i.e.*, prostratin, PMA and bryostatin) as well as PHA, TNF-α and the 6 µM concentration of MRK-1. This pattern of compound clustering was supported when data from the QVOA was included and a reduced compound set analyzed (Figure 2B).

It is noteworthy that HMBA clustered interspersed with the HDAC inhibitors, which suggests potential similarities in the mechanism of action. The recent finding that the HDAC inhibitor, SAHA, can release P-TEFb from the inhibitory 7SK snRNP complex [70] provides a potential explanation for the close clustering of HMBA and HDAC inhibitors. In fact, a provocative finding in that study was that the viral reactivating ability of SAHA did not correlate with histone H3 or tubulin acetylation but, rather, with release of P-TEFb [70].

As shown in Figures 2A and 2B, the NFκB agonists PMA, prostratin, bryostatin, PHA and TNF-α cluster together. This result indicates that NFκB agonists consistently work as latency-reversing drugs across the different models, and that NFκB may play a central role in viral reactivation from latency, independent of the model used. In agreement with that, PHA and PMA were active in all the models tested.

In summary, the clustering of compounds based on their activation of HIV across models conforms to what would be expected biologically and validates the analytical approach utilized in the current study.

## Discussion

This study represents the first experimental comparison among several broadly used HIV latency systems, including primary cell models, transformed cell lines and patient-derived cells. To establish these comparisons in an unbiased manner, we chose a panel of known stimuli that were tested in parallel in the selected cell models. The methodology was designed to circumvent variations due to batch, formulation or concentration differences in the compounds tested. To the extent possible, the duration of exposure to each stimulus, the inclusion of appropriate controls and the maximal-response stimulus were standardized as well.

PHA was the only stimulus that uniformly reactivated latent viruses in all systems tested. Most T cells, whether transformed or primary, express CD3ε or CD2, both of which are triggered by PHA. Unfortunately, the therapeutic potential of agonists of the CD3/CD28/CD2 signaling pathway is uncertain, given the plethora of undesirable side effects, including transient lymphopenia, previously observed in patients treated with OKT3 antibodies [71], [72]. PMA also reactivated viruses across models. Responsiveness to PMA was roughly, although not exactly, paralleled by responsiveness to the other PKC agonists tested, prostratin and bryostatin. For example, patient cells were responsive to PMA and prostratin, but not to bryostatin. Differences may be explained by the repertoire of PKC isoforms that is activated by each PKC agonist. This issue will require further exploration, as it is likely that certain PKC isoforms may be more involved than others in the reactivation of latent HIV. It is also plausible that certain PKC isoforms may be able to mediate viral activation with only minimal induction of cellular activation and/or proliferation, which, if true, would clearly be desirable in an eradication strategy.

The addition of Ionomycin to PMA generally provided an enhancement of the activity observed with PMA alone, with the exception of cells in the Spina model. This is intriguing, and contrary to expectations. Ionomycin induces calcium influx, which activates the calcineurin phosphatase that, in turn, activates NFAT. A possible explanation for the loss of activity with PMA+Ionomycin in the Spina model might be the onset of apoptosis, due to a high level of stimulation. However, this was not the case; increased cell death was not observed in these cultures during testing. Virus reactivation in the Spina model was measured by levels of *tat* mRNA transcription after 24 hrs. following exposure to stimulus. In other studies, in which HIV reactivation was tracked by production of soluble p24, virus replication was detected readily 4–5 days after PMA+Ionomycin stimulation (C.A.S., unpublished results). Because PMA+Ionomycin stimulation delivers such strong and immediate cell activation signals, it may be possible that at early time points, limited “signaling resources” in primary T cells could be redirected away from the viral LTR and initiation of *tat* transcription [39]. Additional studies will be necessary to address this mechanistic point.

The activities of cytokines are usually dependent on the presence or absence of their respective receptors on the target cells. TNF-α showed remarkable activity in several J-Lat clones and in patient cells, but was inactive or had low (Lewin) to moderate (Spina) activity in the primary cell models. As stated above, the TNF-R was not found in cultured or fresh T_CM_. Therefore, the high level of responsiveness in patient cells may underlie upregulation of the receptor under the culture conditions utilized, including perhaps the incubation with TNF-α itself. It will be informative to ascertain whether such upregulation occurs, and the specific conditions influencing it. This putative upregulation of the TNF-R is potentially exciting because, if appropriately targeted to cells in the latent reservoir, it would render cells exquisitely responsive to TNF-α or an agonist thereof. Responsiveness to TNF-α clusters among PKC agonists (Figures 2A and 2B), which likely reflects the fact that both types of stimuli culminate in NFκB activation. However, in the analysis displayed in Figure 2A, TNF-α clusters closest with MRK-1, an HDACi, at 6 µM.

HDAC inhibitors are the first drug class to be utilized in clinical trials for HIV eradication and the results so far have been promising [69] because intracellular increases in HIV transcription were induced *in vivo* during SAHA treatment. Future development of HDAC inhibitors should be directed at ascertaining which HDAC isoforms are more involved in maintaining HIV latency, so that they can be specifically targeted.

In general, the Lewin model clustered closely with the J-Lat 5A8 cells and both of these clustered with the patient cell outgrowth assay. However, one of the major differences between both models pertains to responsiveness to the HDACi, MRK11, which blocks Class II enzymes. Cells in the Lewin model displayed very high sensitivity to all tested HDACi, and were the only ones in this study to exhibit a substantial response to MRK11. In contrast, patient cells in the outgrowth assay did not respond to MRK 11. Three primary cell models, Greene, Planelles and Siliciano, had extremely low or no sensitivity to HDACi treatments. It is unclear what aspects of the biology of the cells or the latent viruses in these models renders the latent viruses so refractory to the effects of HDAC inhibition. As we suggest below, the low levels of active P-TEFb components in resting cells may constitute a major barrier to efficient transcription, which may not be overcome simply by inhibition of HDACs. Recent observations indicate that incubation of primary resting cells with stimuli that induce P-TEFb allows the cells to then become responsive to HDAC inhibition (Matija Peterlin, UCSF; personal communication).

Cells in the Lewin and patient cell/QVOA models shared responsiveness to HMBA, while most other models had very low or no responsiveness to this agent. HMBA facilitates the dissociation of P-TEFb from the 7SK snRNP complex and makes P-TEFb more readily available to interact with Tat, and then to be recruited to the TAR loop on nascent viral RNAs. This is an early step in the transcriptional activation of the silent provirus and, therefore, it is viewed as a “gate keeper” step. Recent reports [18], [70], [73] have suggested that resting T cells contain very low levels of cyclin T and phosphorylated CDK9, leading to the hypothesis that the activity of the P-TEFb complex is inherently low, and not controlled by recruitment to the inactive 7SK snRNP complex. In view of these observations, Budhiraja et al. explained the lack of responsiveness of cells in the Planelles model as a result of the low levels of cyclin T and of CDK9 phosphorylation [73]. However, the previous model does not explain two of the observed responses in the present studies. First, patient cells and those in the Lewin model responded strongly to HMBA, while also being quiescent. Future studies should be undertaken to test the levels of P-TEFb in these model systems and examine the correlation between levels of P-TEFb and sensitivity to HMBA. Second, J-Lat cells seemed unresponsive to HMBA, while they would be expected to have high levels of active P-TEFb, given that they are dividing cells. We speculate that P-TEFb is not limiting in J-Lat cells, and that the rate-limiting step to active proviral transcription is either at the transcription initiation level, prior to the participation of Tat, or downstream of P-TEFb recruitment. A plausible mechanism for the lack of activity of HMBA in J-Lat cells is through transcriptional interference imposed by a proximal cellular promoter, as was shown for certain J-Lat clones, including J-Lat 6.3 and 8.4 [74].

Ionomycin was a poor inducer of reactivation in all primary cell models and patient cells, and had no detectable activity in the J-Lat cells. Calcium influx is necessary for activation of the NFAT transcription factor, but is not sufficient by itself for optimal viral reactivation. It appears that the full effect of NFAT on HIV reactivation, at least in cultured T_CM_, requires an additional signal provided by LCK activation [15].

PKC agonists were generally potent reactivators in most models tested here. Bryostatin is of particular interest because it stands as the only PKC agonist that is FDA approved and, consequently, data on its pharmacokinetics and toxicity in humans are available [75], [76]. Bryostatin has been tested in clinical trials for cancer and Alzheimer's disease [75], [76]. In addition, bryostatin was shown to synergize with the HDACi, valproic acid, in reactivation of latent HIV in a J-Lat model [76]. Although bryostatins are emerging as potential therapeutics for HIV eradication, they typically induce cellular activation, proliferation and secretion of pro-inflammatory cytokines. Thus, future research will need to identify analogs with diminished capacity to induce such undesirable cellular effects, while preserving the ability to reactivate latent HIV.

No single experimental system of HIV latency completely recapitulated responsiveness to all types of stimuli tested here. The Lewin *in vitro* model displayed the broadest responsiveness. Similarities between responsiveness of patient-derived cells and the Lewin model cells were observed more frequently than with any other model. However, several notable differences separated the previous two models. These were: the high responsiveness of the Lewin model to MRK-11 and bryostatin, which contrasted with the lack of responsiveness of patient cells; and the lack of response of Lewin cells to αCD3/αCD28. The lack of responsiveness of the Lewin model cells to IL-2+IL-7 contrasted with the high responsiveness of the Spina and Siliciano models. Therefore, secondary screening of latency reversing drugs obtained through high throughput systems could be accomplished by using a combination of testing in the Lewin system plus a system that shows complementary properties, such as the Spina or the Siliciano models.

The site of proviral integration can modulate the levels of viral transcription and has been proposed as a mechanism to explain latency [77], [78]. Specifically, integration in the vicinity of actively transcribed cellular genes can lead to transcriptional interference effects [74], [79], [80]. The present study did not attempt to analyze the influence of integration on proviral latency status. However, in a separate study [81], the influence of host cell gene transcription on proviral latency was analyzed and compared for five different models of latency including the Siliciano [17] and Planelles [15] models, a Jurkat model with polyclonal integration [78], infection of primary resting CD4^+^ T cells [82], and infection of primary activated CD4^+^ T cells [82]. When the influence of positioning in the chromosome (regardless of orientation) was examined, proviruses integrated in nearby positions shared the same latency status more often than predicted by chance. However, this trend was only statistically significant when comparing proviruses within each model, but not when comparing proviruses across models. This was interpreted by the authors to mean that local chromosomal features affecting latency are model-specific. Regarding proviral orientation with respect to cellular genes, the Siliciano model exhibited a modest, but statistically significant preference for latent proviruses to be in the same orientation as proximal cellular genes, confirming a previous report [80]. In contrast, the other models exhibited no statistically significant deviation from 50% of latent integrations being in the same orientation as cellular genes.

Rational design of drugs to target HIV latency is not possible at the moment, because we do not have precise knowledge of all the cellular factors and activation pathways that impact viral transcription, leading to productive replication. A second obstacle to rational drug design for viral eradication lies in the notion that while the desired compound should trigger HIV reactivation, it should induce minimal or no cellular activation/proliferation. Therefore, drug screening studies should include an evaluation of the ability of candidate compounds to induce expression of cellular activation markers and proliferation.

## Methods

### Ethics statement

Studies involving human peripheral blood mononuclear cells were conducted at the following institutions, and approved by the respective internal boards as indicated:

#### University of California San Diego and the Veterans Administration San Diego Healthcare System

This project was reviewed and approved by the IRB of the Human Research Protections Program of the University of California San Diego (protocol #111173, 8/16/2012). Only adult subjects were recruited into the study and they provided written informed consent.

#### Monash University and Alfred Hospital

This project was approved by the Monash University Human Research Ethics Committee, project number 2012000032, Chief investigator Prof Sharon Lewin. Date of approval, 16 January 2012.

#### University of North Carolina at Chapel Hill

The human studies protocol was reviewed and approved by the Biomedical institutional review board of UNC Chapel Hill. All adult subjects provided written informed consent. University of Utah. Our studies used blood purchased from the American Red Cross without subject identifiers. Therefore, the University of Utah Internal Review Board considered our work on this project exempt from further protocol review and approval.

#### University of California San Francisco and Gladstone Institute of Virology

Since blood was purchased from the blood bank without subject identifiers, the UCSF Committee on Human Research considered our work on this project exempt from further protocol review and approval.

#### Johns Hopkins University School of Medicine

Blood was obtained from healthy donors through a protocol approved by the Johns Hopkins University School of Medicine Internal Review Board #4. All study subjects provided written informed consent prior to participation in the study.

### Cell Models of HIV Latency

#### Greene Model, primary T cells

Healthy PBMCs were isolated from leukoreduction system chambers (Trima Accel System, Terumo BCT, Inc.) by Ficoll-Hypaque density gradient centrifugation. Total CD4+ T cells were immediately isolated by negative selection using EasySep Human CD4+ T Cell Enrichment Kit (Stemcell Technologies). Isolated CD4+ T cells were plated at a density of 1×10^6^ cells per well in a 96-well v-bottom plate at a volume of 200 µL RPMI containing 10% FCS. Cells were spinoculated with 100 ng (p24Gag) of NL4-3-Luciferase at 2,500 rpm for 2 h at 37°C. After spinoculation cells were resuspended at a cell density of 1×10^6^ cells/mL in RPMI and 10% FCS containing 5 µM saquinavir mesylate (Sigma-Aldrich) and incubated at 37°C for 72 h. Infected cells were then plated at 1×10^6^ cells/well in a 96-well flat-bottom plate in 200 µL RPMI and 10% FCS containing 30 µM raltegravir (Santa Cruz Biotechnology). The compounds to be tested were immediately added at the indicated concentrations, and cells were incubated at 37°C. To analyze these samples, cells were washed with PBS and lysed in Glo Lysis Buffer (Promega). Luciferase activity was quantified using a BD Monolight Luminometer after mixing 50 µL of lysate with 50 µL of substrate (Luciferase Assay System, Promega). Relative light units were normalized to protein content determined by BCA assay (Pierce). All compounds were tested with cells from 3 different donors and triplicate samples per assay. When maximal stimulation was used, cell viability was over 75% as determined by flow cytometry.

#### Lewin Model, primary T cells

Peripheral blood mononuclear cells (PBMC) were isolated from buffy coats obtained from the Australian Red Cross Blood Service (Southbank, Australia). Latently infected resting CD4+ T cells were prepared and reactivated with a panel of agents, as previously described with modifications [21], [24]. Briefly, resting CD4+ T cells were isolated, rested in culture 24 hours, incubated with the chemokine CCL19 at 29 nM (R&D, Minneapolis, MN) a further 24 hours and then infected with the CXCR4 using virus HIV-1 NL4-3 at 0.5–1 CPM reverse transcriptase activity per cell for 2 hours. Cells were washed and cultured in RF10 and 1 IU/ml IL-2 for 4 days to establish latency. Establishment of latency was confirmed by detecting integrated HIV DNA using a nested Alu-LTR PCR [83] and limited or no production of reverse transcriptase (RT) in cell culture supernatant. To induce virus production, CCL19-treated latently infected CD4+ T-cells were plated at 0.3×10^6^ cells/well in a 96-well plate on day 4 post infection and incubated with the test compounds. PHA-activated, CD8-depleted feeder PBMC were added 24 hours after the activating stimulus at a ratio of 2 feeders per T cell to amplify virus replication, as previously reported [46]. A half media change was performed 3 days post activation. Virus production was measured in supernatant by quantification of RT production at 5 days post activation. All compounds were tested with cells from 4 different donors and triplicate repeats per assay. Routinely, prior to stimulation in this assay, viability was greater than 80%. Viability was not assessed at later time points due to the addition of feeder cells.

#### Margolis, patient cells/viral outgrowth assay (QVOA)

For experiments using primary cells obtained from HIV-infected, ART-treated, aviremic patients, outgrowth assays were performed, as described previously [22]. Lymphocytes were obtained by continuous-flow leukapheresis from HIV-infected volunteers receiving stable ART with plasma HIV-1 RNA less than 50 copies/ml and a CD4+ T cell count of more than 300 cells/ml. Patient cells were incubated with compounds for 24 hours to disrupt latency; the exception to this was combined signaling with IL-2 and IL-7, which was maintained in cultures for the first 2 days of the assay. Following this initial incubation, the patient cells were plated in replicate dilutions and stimulated with PHA-L, allogeneic irradiated PBMC from a sero-negative donor, and rIL-2. CD8-depleted PBMC, collected from the same HIV sero-negative donor, were added to culture at regular intervals. Assays were carried out to 15 days of co-culture, at which time soluble HIV p24 Gag antigen was measured by ELISA; any cultures with detectable levels of p24 were deemed to be potentially positive. Cultures were continued to day 19, and only cultures that maintained an equivalent or greater level of p24 antigen on day 19 as on day 15 were scored as positive. In duplicate experiments, the same patient's cells were tested using cells from the same sero-negative donor. All compounds were tested with cells from 3 different HIV-infected patients. To minimize the effects of the variance of the assay and of the varied frequency of resting cell infection on the response to a stimulus, the results from the limiting dilution cultures from all 3 patients were pooled to calculate one common IUPM value. The IUPM values thus obtained were then normalized to that obtained with PHA incubation. Therefore, standard deviations for data in the QVOA could not be calculated.

#### Planelles Model, cytokine-polarized primary T cells

Latently infected primary T cells were generated, using a published method [14], [15]. Briefly, CD4+ T cells, with a naïve phenotype were isolated by negative selection from fresh peripheral blood and exposed to αCD3 plus αCD28 antibody-coated beads (Dynal, 1∶1 bead∶cell ratio) for 3 days, with the addition of TGF-β (10 µg/mL) and αIL-4 (1 µg/mL) and αIL-12 (2 µg/mL) monoclonal antibodies. Cells were then cultured in rIL-2 for an additional 4 days to derive a differentiated “non-polarized” subset (NP), which is considered the *in vitro* equivalent of T_CM_ [14], [25], characterized by expression of CCR7, CD27, CD45RO and the IL-7 receptor (CD127). At this point, latent infection was established through spinoculation with an infectious HIV-1 clone, defective in *env*. This virus was produced by co-transfection with an HIV-1 X4 *env* expression vector in 293T cells (DHIV). At one week following infection, most productively infected cells were lost through apoptosis, and only latently infected and uninfected cells remained. To maintain viability, cells were cultured with rIL-2 for the duration of the experiments. Reactivation of HIV was measured by intracellular expression of Gag (p24), using flow cytometry analysis. All compounds were tested with cells from 4 different donors and triplicate repeats per assay. Viability at the time of readout was between 69 and 94% for all treatments.

#### Siliciano Model, Bcl-2 transduced primary T cells

The model characteristics were described previously, in detail [17]. To summarize, bulk primary CD4+T cells were isolated by positive selection (MACS) from peripheral blood, and stimulated with immobilized αCD3 antibody in microplates in the presence of αCD28 antibody and rIL-2 (100 U/mL). After 3 days, the activated cells were transduced with the lentiviral vector, EB-FLV, for constitutive expression of Bcl-2, and then expanded in culture with rIL-2 for an additional 3 days. The transduced cells returned to a resting state after 3–4 weeks of culture in the absence of any exogenous cytokines. At this point, viable cells were recovered and re-stimulated with immobilized αCD3 antibody+rIL-2, as before. After 10–12 days, the Bcl-2 transduced cells were infected with the HIV reporter construct NL4-3-Δ6-drEGFP (Δ*env*; mutated *gag*, *vif*, *vpr*, *vpu*, *nef*) packaged in trans with X4-tropic HIV-1 Env. The infected cells were cultured with rIL-2 for 3 days, and then maintained for at least 4 weeks in the absence of exogenous cytokines. To recover latently infected CD4 cells, the GFP-negative portion of the culture was purified by flow cytometry sorting. The isolated cells had a predominant quiescent cell phenotype (G_0/1a_) that is representative of effector memory T cells (T_EM_), with expression of CD45RO^+^RA^dim^/CCR7^−^/CD25^dim^ [17]. Virus reactivation was measured by expression of GFP, using flow cytometry analysis. All compounds were tested with cells from 3 different donors and duplicate samples per assay. After exposure to the test compounds, the cell viability at the time of assay harvest was between 70–84% for all conditions, except for the following treatments: highest PHA concentration, 41%; highest concentration of all HDACi, 51–62%; Ionomycin, 63%.

#### Spina Model, primary T cells

Primary CD4+T cells were isolated by negative selection (RosetteSep) from peripheral blood. A portion of the cells was maintained in culture, without stimulation, for 4 days. Another cell aliquot was stained with CFSE dye, infected with replication-competent NL4-3, and cultured in microplates with immobilized αCD3 and αCD28 antibodies to induce cell proliferation and productive HIV replication [27]. After 4 days of culture, the CFSE-stained, infected and proliferating cells were removed from the αCD3/αCD28 stimulus and mixed with the unstimulated, uninfected autologous CD4 cells at a ratio of 1∶4. The co-culture was maintained for 3 days with the addition of exogenous rIL-2 (5 U/mL). On day 7, the non-dividing “bystander cells” were isolated from co-culture by flow cytometry sorting, and gating on the small undivided, CFSE-negative subpopulation (FSC vs. CFSE). The recovered infected bystander cells (90–95% viable) were cultured in fresh medium, in the absence of any exogenous cytokines, for 2 additional days, before being used in subsequent experiments. HIV reactivation was performed in the presence of the integrase inhibitor, raltegravir (0.1 mM) to exclude any contribution from unintegrated virus. The latently infected population ranged from 1 to 12% of cells containing integrated HIV DNA [84], depending on the cell donor, and 0.5–5% of cells with replication competent provirus that is inducible following maximal stimulation with immobilized αCD3/αCD28 antibodies, as measured by expression of intracellular Gag. Reactivation of HIV was measured by RT-qPCR quantification of cell-associated *tat* RNA and normalization to number *tat* copies per 10^8^ copies 18S RNA. All compounds were tested with cells from 3 different donors and duplicate repeats per assay. After exposure to the test compounds, the cell viability at the time of assay harvest was between 75–96% for all conditions.

### Panel of Cell Stimuli

The test compounds, listed in Table 2, were obtained, and stocks prepared and distributed centrally to each of the participating laboratories by the CARE Pharmacology Core of the University of North Carolina. The compounds were tested in each cell model at the following final concentrations: αCD3/αCD28-conjugated beads (Dynal) at 1∶1 bead∶cell ratio; PHA-M (Sigma) at 1.1, 3.3, 10 µg/mL; PMA (Sigma) at 2 nM for primary T cells, 16 nM for J-Lat cells; Ionomycin (Sigma) at 0.5 µM; prostratin (LC Laboratories) at 0.3, 1, 3 µM; bryostatin (provided by the National Cancer Institute) at 10, 33, 100 nM; SAHA/vorinostat (Merck) at 0.11, 0.33, 1 µM; MRK-1 (class I HDACi, Merck) at 0.67, 2, 6 µM; MRK-11 (class II HDACi, Merck) at 3, 10, 30 µM; HMBA (Sigma) at 0.3, 1, 3 mM; TNF-α (Peprotech) at 10 ng/mL; IL-2 (Peprotech) at 30 IU/mL; IL-7 (Peprotech) at 50 ng/ml. IL-2, IL-7, TNF-α, and αCD3/αCD28 bead stocks were prepared in RPMI culture medium; HMBA stock was prepared in water. All the other compounds were prepared in DMSO solvent. Unless otherwise specified, each cell model tested and the HIV outgrowth assay included the controls: untreated (base culture medium), 0.1% DMSO, 0.5% DMSO (specific to 10 µg/mL PHA). The exposure time of cells to compounds was standardized across the models to 24 hrs., except for PHA (48 hrs.), αCD3/αCD28 beads (48–72 hrs.), and IL-2+IL-7 (5 days). The timing of assay read-outs for HIV reactivation was specific to each model system, dependent on unique cellular and viral characteristics.

### Data normalization, heatmap visualization and clustering

Initially any compound or any concentration of a compound that was not used universally across all models was removed. The untreated control, representing background activation, was subtracted from each compound for each donor in each model. Activation values for each compound were then averaged across donors within each model and any activation resulting from the DMSO condition was subtracted from those compounds that were dissolved in DMSO. DMSO has structural similarity to HDAC inhibitors, as some of these compounds were derived from DMSO following the observation of DMSO effects on transformed cells [85]. Average activation values for each compound were then normalized within each model by dividing by the average activation value for the highest concentration of PHA used so that models could be compared to each other. Finally, examining the distribution of average activation values across compounds revealed right-skewed data for each model and thus a log_10_ transformation was performed. Constants were added to the average activation value for each compound to account for negative values prior to log_10_ transformation and to shift activation values into a range that reflected their actual activation level.

An unsupervised approach was used to determine the relationship between compounds based on their ability to activate HIV across models and also between models based on their response to compounds. Cluster 3.0 [86] was used for hierarchical clustering of compounds and models such that distances were calculated using the Euclidean based metric and then clustered using the average linkage method. The results were visualized in a heatmap using Java TreeView [87]. The statistical significance associated with clustering was determined using pvclust [88] (R package), which calculates approximately unbiased (AU) *p*-values that are computed using multiscale bootstrap resampling such that 95% equates to a *p*-value cut-off of 0.05. These normalization procedures and hierarchical clustering approaches were performed twice since not every compound was assessed at every concentration in the QVOA model. Specifically, the were performed once using a complete list of compounds but without data from the QVOA and a second time with a subset of compounds but now with the inclusion of data from the QVOA.

### TNF receptor surface analysis

TNFR surface expression was determined using anti-human TNFRI-APC (R&D Systems, Minneapolis, MN). Briefly, 1×10^5^ cells were incubated with 1∶100 anti-human TNFRI-APC in 100 µl of PBS/3%FBS Buffer during 30 min at 4°C followed by flow cytometric analysis in a BD FacsCanto II flow cytometer using the FACSDiva software (Becton Dickinson, Mountain View, CA). Data was analyzed with FlowJo (TreeStar Inc., Ashland, OR).

## Supporting Information

Figure S1 Levels of HIV induction are plotted for each stimulus tested in each cell model. Within each model, results have been normalized to the maximal response of a positive control stimulus (e.g. αCD3/αCD28, PHA, or PMA+Io). The highest level of response for each stimulus is shown, independent of the stimulus concentration associated with the response. Positive responses are rounded to the nearest decile percentage. Open bars indicate a response below 5%.
(TIFF)

Figure S2 TNF-α receptor is not expressed in freshly isolated memory CD4+ cells and is extremely low in cultured central memory cells. Gray curves and black lines represent cells stained with isotype control and TNFR antibody, respectively. J-Lat clone 10.6 was used as a positive control.
(TIFF)
