# Supplementary figures and images for: An In-Depth Comparison of Latent HIV-1 Reactivation in Multiple Cell Model Systems and Resting CD4+ T Cells from Aviremic Patients
Source: PLoS Pathog. 2013 Dec 26;9(12):e1003834. doi: 10.1371/journal.ppat.1003834 (PMC3873446; doi:10.1371/journal.ppat.1003834)

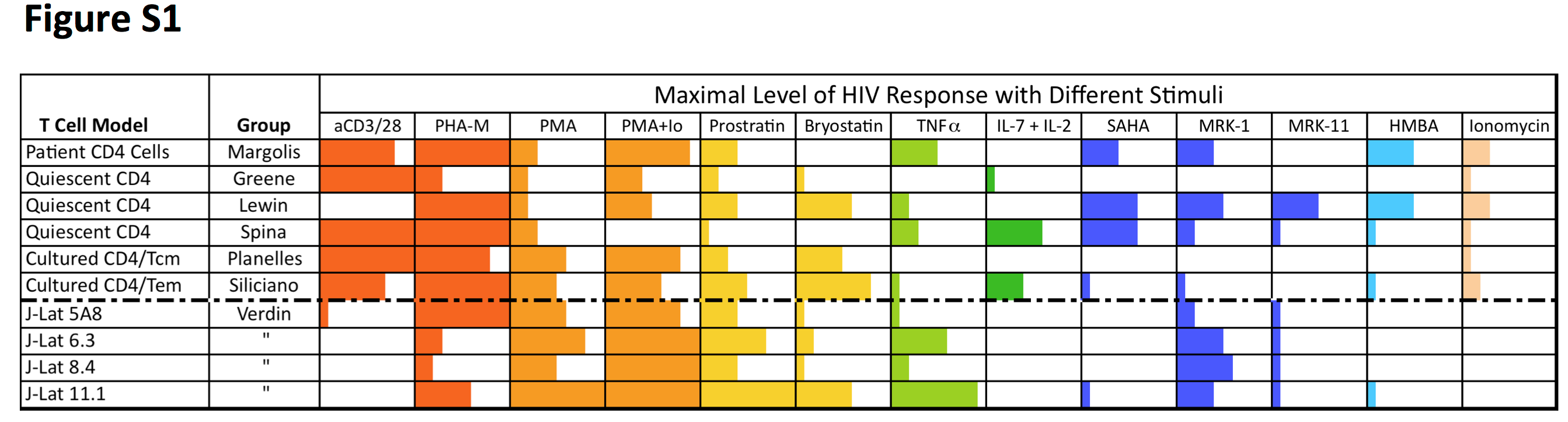

Supplement: Figure S1 — Levels of HIV induction are plotted for each stimulus tested in each cell model. Within each model, results have been normalized to the maximal response of a positive control stimulus (e.g. αCD3/αCD28, PHA, or PMA+Io). The highest level of response for each stimulus is shown, independent of the stimulus concentration associated with the response. Positive responses are rounded to the nearest decile percentage. Open bars indicate a response below 5%. (TIFF) [file ppat.1003834.s001.tiff]

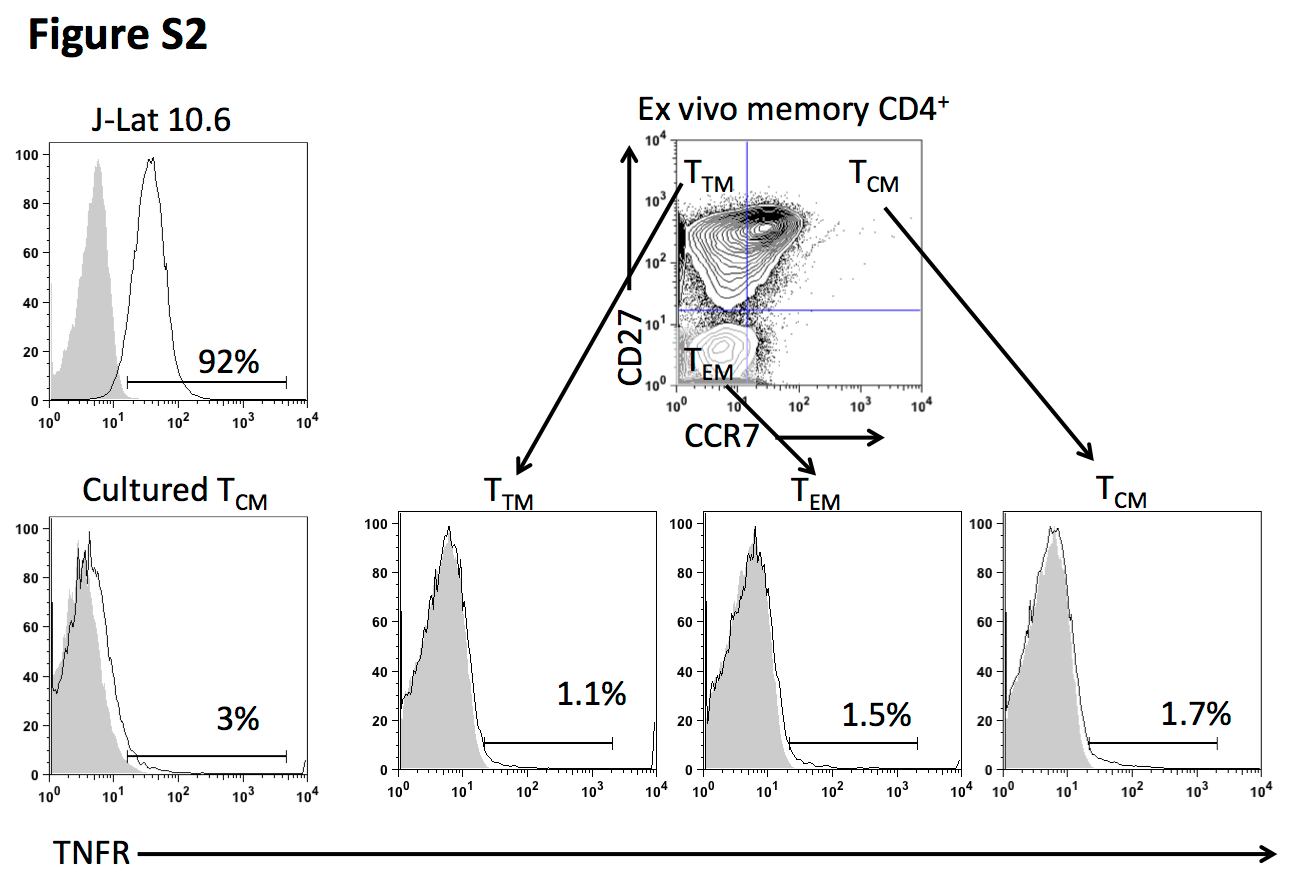

Supplement: Figure S2 — TNF-α receptor is not expressed in freshly isolated memory CD4+ cells and is extremely low in cultured central memory cells. Gray curves and black lines represent cells stained with isotype control and TNFR antibody, respectively. J-Lat clone 10.6 was used as a positive control. (TIFF) [file ppat.1003834.s002.tiff]
